# Sleep as a protective factor of children’s executive functions: A study during COVID-19 confinement

**DOI:** 10.1371/journal.pone.0279034

**Published:** 2023-01-11

**Authors:** Matthieu Beaugrand, Christophe Muehlematter, Andjela Markovic, Valérie Camos, Salome Kurth

**Affiliations:** 1 University of Fribourg, Department of Psychology, Fribourg, Switzerland; 2 University Hospital Zurich, Department of Pulmonology, Zurich, Switzerland; University of Oxford, UNITED KINGDOM

## Abstract

Confinements due to the COVID-19 outbreak affected sleep and mental health of adults, adolescents and children. Already preschool children experienced acutely worsened sleep, yet the possible resulting effects on executive functions remain unexplored. Longitudinally, sleep quality predicts later behavioral-cognitive outcomes. Accordingly, we propose children’s sleep behavior as essential for healthy cognitive development. By using the COVID-19 confinement as an observational-experimental intervention, we tested whether worsened children’s sleep affects executive functions outcomes 6 months downstream. We hypothesized that acutely increased night awakenings and sleep latency relate to reduced later executive functions. With an online survey during the acute confinement phase we analyzed sleep behavior in 45 children (36–72 months). A first survey referred to the (retrospective) time before and (acute) situation during confinement, and a follow-up survey assessed executive functions 6 months later (6 months retrospectively). Indeed, acutely increased nighttime awakenings related to reduced inhibition at FOLLOW-UP. Associations were specific to the confinement-induced sleep-change and not the sleep behavior before confinement. These findings highlight that specifically acute changes of children’s nighttime sleep during sensitive periods are associated with behavioral outcome consequences. This aligns with observations in animals that inducing poor sleep during developmental periods affects later brain function.

## 1. Introduction

The COVID-19 pandemic impacted many aspects of our daily life [1–3]. Many countries implemented a confinement strategy to restrict the dissemination of the virus. This strategy dramatically altered daily routines and had a negative impact on mental health, such that the prevalence of depression and anxiety in some countries nearly doubled [4]. Similar mental health consequences emerged on a global scale as a result of the COVID-19 confinement [5], and low-to-moderate mental health prevalence was reported in up to 50% of the population at the time of COVID-19 confinement [6–8]. Further, in Switzerland the prevalence of self-reported stress levels strongly increased during the most strict phase of confinement between April and November 2020 [9].

Confinement represents a threat to mental health—not only in adults but also in adolescents. Previous government-imposed confinements during epidemics (SARS, Ebola) similarly resulted in increased stress disorders [10], fear, and insecurity [11, 12]. Evidently, the younger population segments, that are still maturing physically and psychologically, are particularly sensitive to the implementation of confinements. For example, in France 14–24 year-olds revealed the highest prevalence of moderate-to-severe depressive symptoms in relation to the COVID-19 confinement [13]. Moreover, in Switzerland the well-being of school-age children was still affected one year after the COVID-19 outbreak [14]. Specifically, families with sparse social support and poor family functioning are at risk for compromised well-being.

However, it remains uncertain whether confinement has long-term negative consequences for development. During confinement-related school closures, as impaired learning progress of school-age children was reported, cognitive performance further declined in preschoolers [15]. As confinement impacted the well-being of families one year beyond the outbreak [14], the chronic effects of confinement on children’s cognition require further investigation. Previous work describing impaired cognitive abilities as a risk to develop mental disorders such as attention disorder [15–17] or depression [18] further emphasizes this necessity.

Besides effects on mental health, alterations in sleep behaviors have emerged from the COVID-19 context in adults, adolescents, and young children. Adults have been shown to experience changes in light exposure, and bedtime regularity, as well as more frequent short sleep nights, worse subjective sleep quality, and increased insomnia [10, 18–23].

Similarly, young children experienced altered sleep, for example, less regular bedtimes, higher day-to-day variability, and fragmentation of sleep [24, 25]. Importantly, changes in sleep habits in children, e.g. delayed bedtimes and shorter sleep duration, were proposed to lead to more psychological problems during confinement [26]. Taken together, the confinement affected sleep in adults and children, yet, whether acutely worsened sleep affects later cognition and behavioral development remains to be clarified.

Sleep fulfills a key role in brain maturation, as evidenced in animal experiments demonstrating developmental consequences of sleep restriction in neurophysiological and cellular processes [26–28]. Studies in humans show that electroencephalographically-assessed sleep depth reflects synaptic homeostasis [29] and predicts anatomical and functional neurodevelopment [30–33]. Accordingly, the electroencephalographic signature of sleep is a marker for children’s cognitive ability [34], and crucially, sleep behavior relates to later behavioral and cognitive development [34–37].

Executive functions characterize higher cognitive processes regulating thought and actions and include planification, decision-making, and inhibition. Thus, executive functions are a strong predictor of school achievement [38, 39]. During childhood, the maturing pre-frontal cortex drives the development of critical executive functions [40–43]. This pre-frontal maturation is understood as a sequential process across multiple cortical regions [44, 45]. Interestingly, brain imaging studies demonstrate that the prefrontal cortex is particularly sensitive to physiological recovery processes that occur during sleep [44, 46, 47]. Accordingly, in adults, acutely shortened sleep or habitually lower sleep continuity are linked with poorer executive functions [48, 49]. Similarly, persisting sleep problems in childhood are associated with later neuropsychological dysfunctioning during adolescence [50]. However, the recovery processes during sleep remain to be fully understood, particularly in the developing brain.

Only few studies investigated the relationship between sleep and executive function during preschool years, for example in the framework of childhood insomnia or chronic disease [51, 52] or by characterizing developmental trajectories of preschool children’s sleep in relation to executive function outcomes at school age [53]. Yet, the existing research entails pure observational data, which is most often the case for research with healthy children, where extensive intervention protocols need to be carefully evaluated in the relation to scientific value generation. It has thus remained unknown, whether the abruptly implemented worsening of preschool children’s sleep, happening for a limited time frame, interacts with executive function outcomes. While longitudinal studies have given important insight in the moderating role of sleep in executive function development [51–53], the global context of COVID-19 confinement now offered a new, unique and ethical opportunity to assess developmental consequences of acutely altered sleep with an experimental-observational study design.

Sleep patterns undergo rapid changes across the developmental period [30], and experimental animal research demonstrates that severe sleep disturbance results in later consequences quantifiable in compromised behavior [27, 53–55]. On the one hand, a diversity of direct and indirect factors can interfere with sleep quality. Thus, while “poor” sleep itself is considered as a developmental risk factor (as outlined above), the quality of sleep is also negatively affected by bedtime irregularity, impaired mental health, or stress from the direct social environment [24]. Stress has been identified as major driving factor in poor children’s sleep concerning parental mental health, but also parenting stress [56, 57].

**Fig 1:**
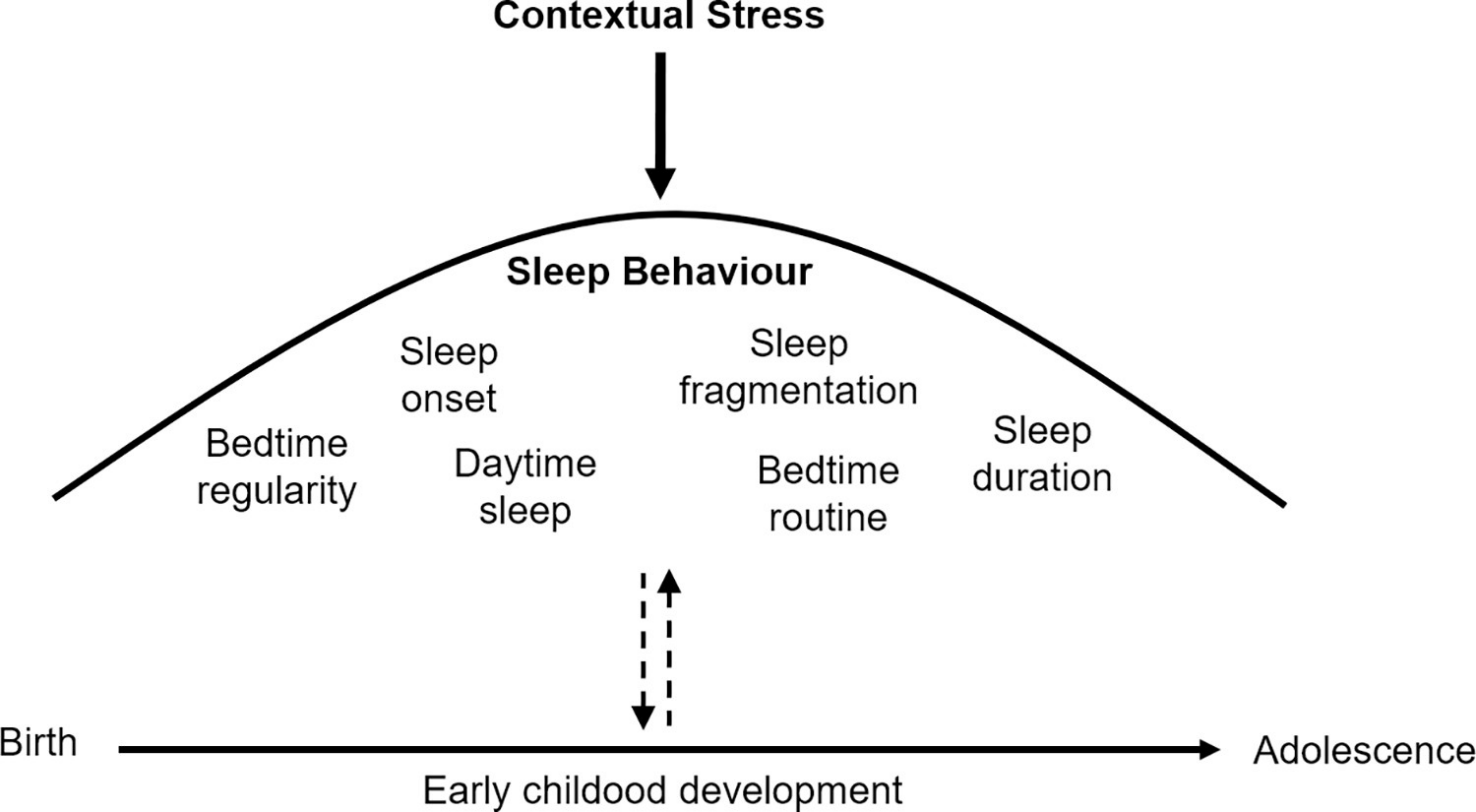
Working model. Sleep behavior is an umbrella protecting developmental outcomes from influences of contextual stress [64–66].

On the other hand, sleep can also be a protective factor. For example, efficient sleep may reduce the adverse consequences of family stress on cognitive abilities [58]. Nevertheless, this interesting concept that preschool children’s sleep quality can be targeted for protecting developmental cognitive processes under challenging circumstances remains understudied. Factors linked to increased quality of children’s sleep include physical exercise [59–61], parental education [62, 63], household structure (i.e., presence of siblings or pets), and caregiver’s engagement in childcare or mindfulness techniques [24]. The existing evidence inspired our working model which considers the quality of sleep as an umbrella protecting developmental outcomes from influences of contextual stress (Fig 1).

The Sleep-Umbrella-Model is the visualization of the concept that maintaining beneficial sleep patterns during periods of contextual stress provides a protecting framework for neurodevelopment. Unfavorable sleep behaviors create a weakness in the protection and thus permit effects of contextual stress to infiltrate. The present study tested the Sleep-Umbrella-Model by examining an observational-experimental setting defined by the COVID-19 confinement. By treating confinement as an observational-experimental intervention, this study investigated whether confinement-induced changes in sleep behavior predict later executive functions in young children. We assessed sleep behavior (*Bedtimes*, *Sleep Latency*, *Nighttime Sleep Duration*, and *Nighttime Awakenings*) with a survey during the confinement, and assessed executive functions 6 months later. We hypothesized that acute worsening of children’s sleep quality (i.e., increased *Nighttime Awakenings* and *Sleep Latency*) relates to reduced executive function scores.

## 2. Materials and methods

### 2.1 Study design

**Fig 2:**
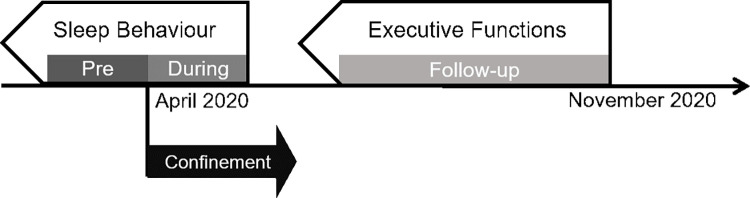
Overview of the study design. During April 2020, the first survey was completed to assess parent-reported sleep of young children during the confinement «during-CONFINEMENT» with the identical questions addressing the time before the confinement «pre-CONFINEMENT». In November 2020, a “FOLLOW-UP” was completed to assess executive functions with a questionnaire referring to children’s behavior retrospectively for the preceding 6 months.

In the COVID-19 confinement period in 2020, participants were recruited through flyers distributed via social media, existing databases, at kindergartens, childcare institutions, and pediatric clinics. To assess children’s sleep during the acute phase of the confinement in April 2020, an online survey was distributed in English, German, French, Italian, and Spanish [67] to be filled out by caregivers. Both a retrospective assessment referring to the time before the confinement (pre-CONFINEMENT), as well as an assessment for the present situation (during-CONFINEMENT) were performed. A follow-up survey in November 2020 (FOLLOW-UP) assessed executive functions in the same participants retrospectively over the preceding 6 months (Fig 2). For both surveys, an English version was translated by the authors into French, German, Spanish, and Italian. For each language, at least two native speakers approved the translation. The study was approved by the institutional ethics board of the University of Fribourg, Switzerland.

Data on 412 preschool children were collected. Participants who did not provide written consent or did not complete both surveys were excluded, resulting in a study population of 45 children, aged 36–72 months (Mage = 53.3 ±4.4 SD, 27 females) living in Europe (30 in Switzerland, 7 in Spain, 2 in France, 1 in Germany, Italy, Austria and Netherlands, 2 unspecified). The condition at this time was similar among represented countries at the two assessment time points.

### 2.2. Sleep behaviour

Children’s sleep was quantified with the Children’s Sleep Habits Questionnaire [68] as part of the first survey (pre-CONFINEMENT and during-CONFINEMENT). We analyzed children’s sleep behavior in 4 core sleep domains (as in [24]: *Bedtime*, *Sleep Latency*, *Nighttime Sleep Duration*, and *Nighttime Awakenings*. Caregivers rated the statements “My child goes to bed at the same time at night.” (*Bedtime*), “My child falls asleep within 20 min after going to bed.” (*Sleep Latency*), “My child sleeps about the same duration each 24-h day” (*Nighttime Sleep Duration*) and “How often does your child wake up during the night” (*Nighttime Awakenings*). Each question was answered twice, for pre-CONFINEMENT (retrospectively) and during-CONFINEMENT, with ratings on a scale from 1 to 5: 1 = never (0 day/week), 2 rarely (1 day/week), 3 sometimes (2–4 day/week), 4 usually (5–6 day/week) and 5 always (7 days/week).

### 2.3. Assessment of executive functions

The Behavior Rating Inventory of Executive Function—Preschool Version (BRIEF-P) was completed by parents in November 2020 (FOLLOW-UP). The BRIEF-P is an established instrument to quantify executive functions from ages 2 years to 5 years and 11 months [69]. Only BRIEF-P parents’ rating was obtained and rating for teachers was not considered due to home-schooling at the time of the survey. Sixty-three statements on children’s behavior were rated retrospectively with a 3-point Likert scale (never, sometimes, always), referring to the behavior during the preceding 6 months. Statements were further grouped into 5 subscales for each participant following the BRIEF-P scoring rules (*Inhibit*, *Shift*, *Emotional Control*, *Working Memory* and *Planning/Organizing*; Table 1).

**Table 1:** Subscales of executive function quantified from the BRIEF-P: Inhibit shift, emotional control working memory, and plan/organize.

Subscale	Specification
Inhibit	Assessment of inhibitory control and impulsivity, i.e. the ability to resist the impulse and the ability to stop one’s behavior when appropriate.
Shift	Quantification of the ability to make appropriate changes to adapt to new situations.
Emotional Control	Measure of the child’s ability to adapt or control emotional responses.
Working Memory	Assessment of the ability to retain information to accomplish a task, encode information, establish goals, plan and sequential steps to achieve goals.
Plan/ Organize	Quantification of the ability to manage present and future tasks. It is composed of two components: planning and organization. The planning component assesses the ability to anticipate future events. The organizing component refers to the ability to order information and identify key ideas or concepts when learning or communicating information.

**Fig 3:**
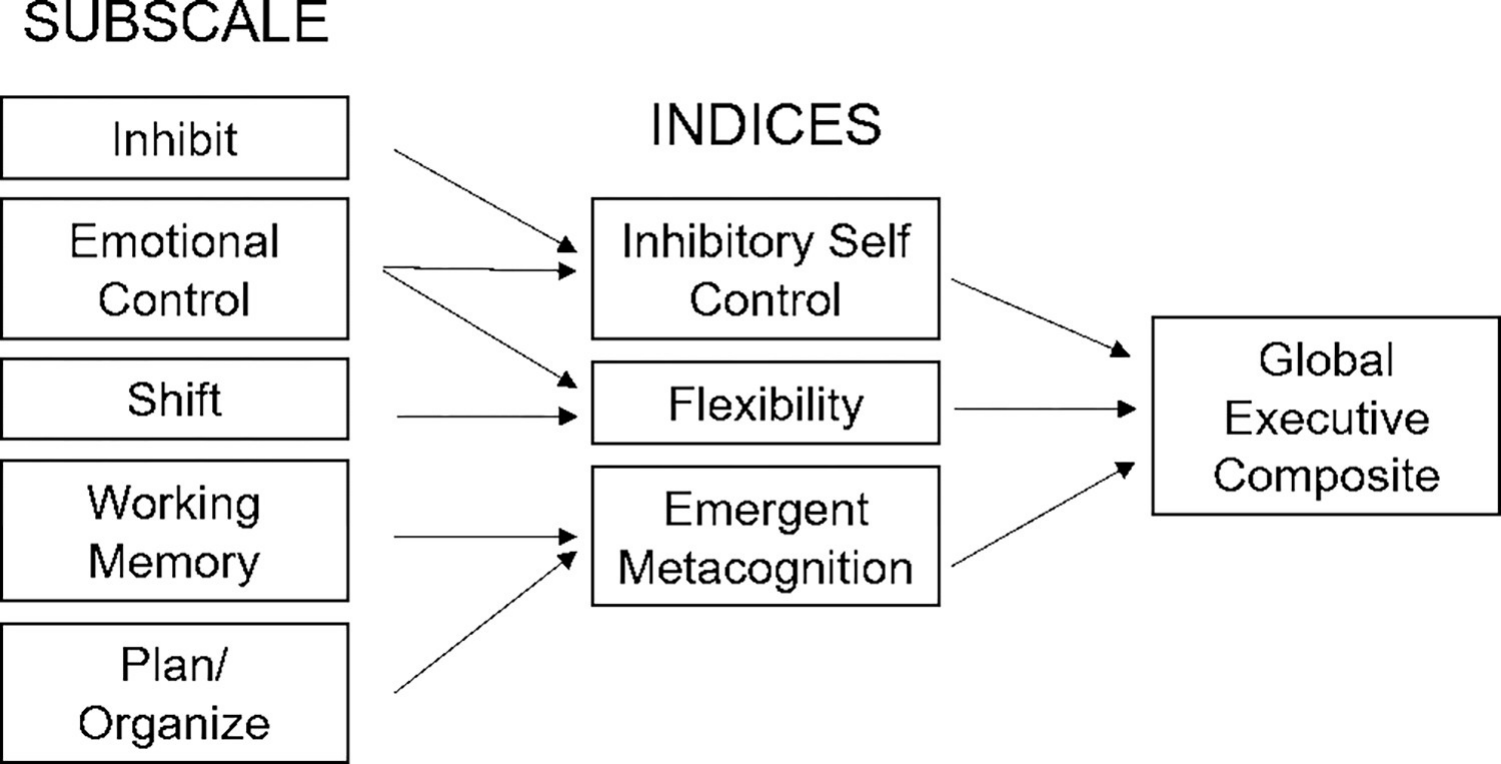
Overview of BRIEF-P standard analysis to quantify executive functions in preschool children, including computation of subscales, indices, and composite score.

From the subscales, 3 indices were computed for each participant to assess *Inhibitory Self Control*, *Flexibility*, and *Emergent Metacognition* (Table 2, Fig 3; standardized computations). Further, the overall score Global Executive Composite (GEC) was calculated across all subscales, to capture global executive functions for each child. As further part of the standard BRIEF-P processing, individual raw scores of the 5 subscales and the 3 indices were transformed into T-scores, based on a normalized table and accounting for age and sex [69].

**Table 2:** Indices of executive function quantified from the BRIEF-P: Inhibitory self control, flexibility and emergent metacognition.

Index	Specification
Inhibitory Self Control	Measure to represent the ability to adapt the child’s actions, behavior, and emotion with appropriate inhibitory control. This index is composed of the subscales Inhibit and Emotional control.
Flexibility	Representation of the ability to act flexibly between actions, behavior, and emotions. This index is composed of the subscales Shift and Emotional Control.
Emergent Metacognition	Quantification of the ability to solve problems through planning and organization while holding ideas and efforts to complete the task in working memory. This index consists of the subscales Working Memory and the Plan/Organize.

For executive functions score processing, two standardized control scales were taken into account. First, the negativity subscale, which determines whether the answer is unusually negative in comparison to a normative population, and secondly, the inconsistency scale, which indicates how similarly the respondent answered to different items of the BRIEF-P [69]. Both control scales were considered in our analysis and none of the participant’s answers fell below these cut-off values, indicating that data were reliably consistent and could thus be included.

### 2.4. Covariates

We considered specific covariates for the analysis, in alignment with previously identified “risk and protective” factors in the confinement context of young children [24], i.e., *caregiver’s level of stress*, *quarantine status*, *time spent on mindfulness strategies and the presence of siblings*. These were defined at the time of the first survey completion and were included in the additional analysis as fixed factors (statistical details follow). Further, we captured children’s behavior reactions to the change of circumstances (*i*.*e*., the pandemic) via parent estimates with the question in the first survey: “Overall, how did the child react to the change of circumstances?”. Possible responses were “good”, “badly”, “neutral”, “angry”, “happy” or “anxious”, which was included in an exploratory analysis.

### 2.5. Statistical analysis

We first evaluated the acute effect of COVID-19 confinement on young children’s sleep as the change in sleep behaviors from pre-CONFINEMENT to during-CONFINEMENT, using a Wilcoxon signed-rank test.

Second, we tested the main hypothesis that the effects of confinement on sleep went beyond acute changes, and affected later executive functions (composite score, indices, subscales). For this purpose, we applied linear mixed models with the fixed factors age, sex and pre-to-during-CONFINEMENT change in sleep behaviors (*Bedtimes*, *Sleep Latency*, *Nighttime Sleep Duration* and *Nighttime Awakenings*), pre-to-during-CONFINEMENT, and subject ID as a random effect to account for inter-individual differences. For each subscale (*Inhibit*, *Shift*, *Emotional Control*, *Working Memory*, *and Plan/Organize*), index (*Inhibitory Self-control*, *Flexibility*, *and Emergent Metacognition)* and the GEC score, the best fitting model was identified separately by backward selection using the Akaike Information Criterion (AIC). All p-values were corrected for multiple testing by means of the false discovery rate [70].

Third, we investigated the impact of covariates (*caregiver’s level of stress*, *quarantine status*, *time spent on mindfulness strategies*, *presence of siblings*) by adding them to the fixed factors of the previous linear mixed models for each subscale, index and the GEC score. The software R and the packages MASS, mice, and nlme were used for computations.

## 3. Results

### 3.1. Sleep Behavior changes during the acute confinement phase

**Fig 4:**
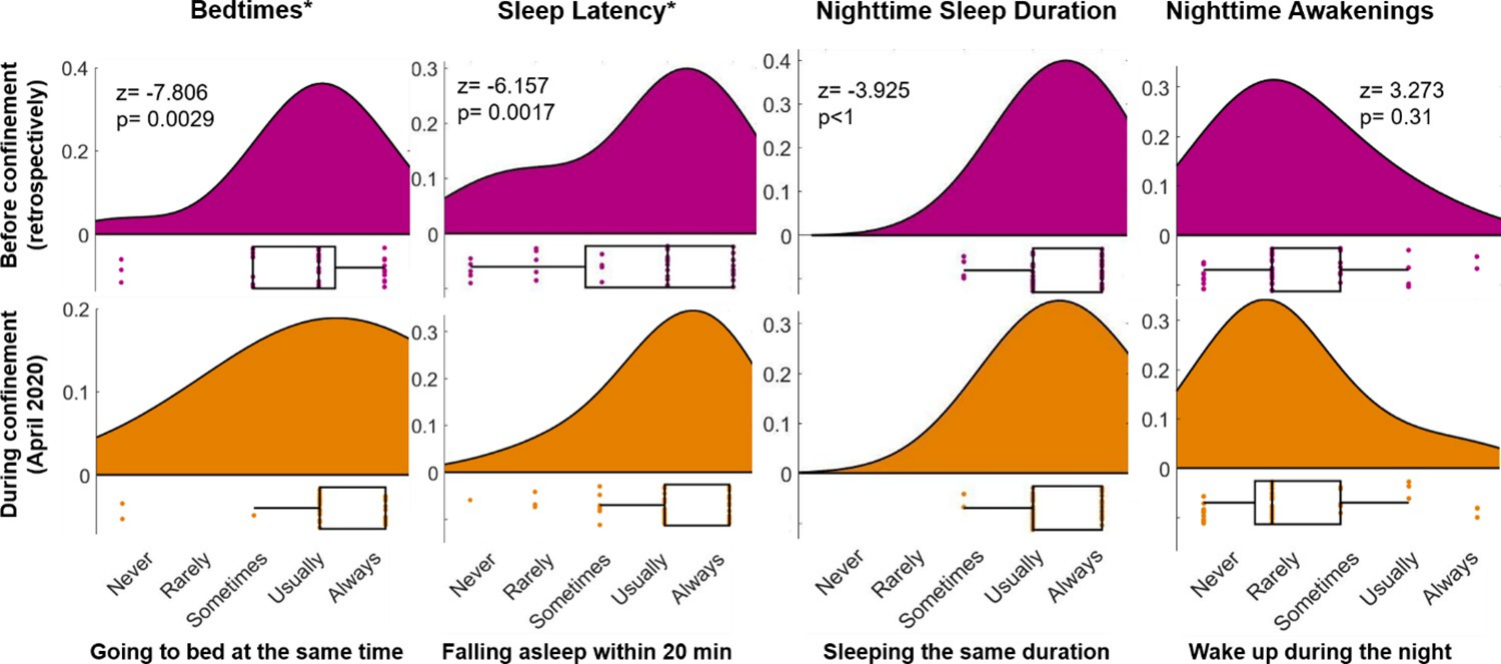
Acute changes in sleep behaviors in preschool children during the COVID-19 confinement. In pink, distribution of sleep behaviors is presented for pre-CONFINEMENT, orange refers to sleep during-CONFINEMENT. Dots represent values of individual subjects. Significance level based on a Wilcoxon signed-rank test is indicated as *p<0.01.

We examined the effect of confinement on preschool children’s sleep by the change from pre-CONFINEMENT to during-CONFINEMENT and observed two major changes (Fig 4). First, bedtimes became more regular illustrating that children were more likely to go to bed at similar clock times every day during the confinement as compared to before (p = 0.0029; z = -7.806; Fig 4). Second, short sleep latency was more often reported, indicating that children fell asleep within 20 minutes more frequently during the confinement than before (p = 0.0017; z = -6.157). No further significant changes were found in children’s sleep behavior, such that neither the stability in *Nighttime Sleep Duration* nor *Nighttime Awakenings* changed as part of the acute confinement implementation.

### 3.2. Changes in sleep behavior predict outcomes of executive functions

The second analysis examined whether preschool children’s sleep before the confinement relates to executive functions outcomes 6 months later. We tested whether (*Bedtimes*, *Sleep Latency*, *Nighttime Sleep Duration*, or *Nighttime Awakenings*) in pre-CONFINEMENT predicted executive functions composite, indices, or subscales (*Inhibit*, *Shift*, *Emotional Control*, *Working Memory*, and *Plan/Organize*) at FOLLOW-UP. No significant effect was found, indicating that preschool children’s *Bedtimes*, *Sleep Latency*, *Nighttime Sleep Duration*, *or Nighttime Awakenings* do not predict cognitive outcomes quantified as executive functions 6 months later (S2 and S3 Figs, BRIEF-P’s Raw and Corrected scores distribution available in S4 Fig).

In a third step, we determined whether the acute change in children’s sleep behavior under the COVID-19 confinement predicted executive functions 6 months later. Thus, we tested whether regularity of *Bedtimes*, *Sleep Latency*, *Nighttime Sleep Duration*, *and Nighttime Awakenings* pre-to-during-CONFINEMENT predicted executive functions subscales *(Inhibit*, *Shift*, *Emotional Control*, *Working Memory* and *Plan/Organize)*, indices (*Inhibitory Self-control*, *Flexibility*, *and Emergent metacognition*) and the GEC at FOLLOW-UP. Indeed, results confirmed that the individual change in sleep behavior during the confinement predicts executive functions, specifically, changes in *Nighttime Awakenings* predicted later *Inhibitory Self-Control Index*.

#### 3.2.1. Effect of age, sex and covariates

While no significant effect of age on the different executive function subscales and composites was observed, this was not the case for sex. Specifically, the models identified sex as a significant predictor of the inhibit score subscale, and particularly, boys had lower inhibitory scores than girls (b = -9.966, p = 0.026). We observed similar effects in *Emotional Control Subscale* (b = -8.466, p = 0.032), *Inhibitory Self Control index* (b = -9.96, p = 0.002), *Emergent Metacognition index* (b = -6.20, p = 0.048), and GEC (b = -8.04, p = 0.014) with boys demonstrating lower scores than girls.

The inclusion of covariates demonstrated that increased *caregiver’s level of stress* predicted reduced *Flexibility* index (b = -5.97, p = 0.004), *Shift* subscale (b = -3.40, p = 0.034) and GEC (b = -3.53, p = 0.016). In addition, the covariate inclusion (caregiver’s level of stress, quarantine status, time spent on mindfulness strategies, presence of siblings) slightly affected results, yet without modifying the significance level for children’s *Nighttime Awakenings* as predictor of *Inhibitory Self Control Index* (b = -4.35, p = 0.01,) *Inhibit* (b = -3.44, p = 0.028) and *Emotional Control Subscales* at FOLLOW-UP (b = -4.08, p = 0.022).

The exploration of children’s behavioral reactions to the pandemic context revealed that only 4 preschoolers reacted negatively (reaction “badly”, “angry” or “anxious”), which corresponds to 8.9% of participants. This information was thus not included in the linear model. Instead, we explored reactions by grouping reactions into “positive” (good, happy; n = 32), “neutral” (neutral; n = 9), or “negative” (badly, angry, anxious; n = 4). The change in sleep regularity was not significantly different among the three groups (M ± SD: 0.28 ± 0.58 for “positive”; 0.11 ± 0.33 for “neutral”; 1.0 ± 1.41 for “negative”; “positive” vs “neutral” p<1; “positive” vs”bad” p<1; “neutral” vs ““bad” p = 0.75, Wilcoxon signed rank test), neither the change of sleep latency (0.41 ± 0.98 for “positive”; 0.33 ± 0.71 for “neutral”; 1.25 ± 1.26 for “negative”; “positive” vs “neutral” p = 0.50; “positive” vs”bad” p = 0.25; “neutral” vs ““bad” p = 0.75). No group effect was found in the change in sleep duration among the reactions (-0.06 ± 0.62 for “positive”; 0.22 ± 0.44 for “neutral”; 0 ± 0 for “negative”—zeros indicate that no change occurred in this variable; “positive” vs “neutral” p = 0.50; “positive” vs”bad” p<1; “neutral” vs ““bad” p = 0.50), nor in the change in awakenings (-0.03 ± 0.59 for “positive”; -0.22 ± 0.44 for “neutral”; -0.50 ± 0.58 for “negative”; “positive” vs “neutral” p<1; “positive” vs”bad” p = 0.50; “neutral” vs ““bad” p = 0.12).

#### 3.2.2. Confinement-induced changes in children’s sleep predict later inhibitory behavior

**Fig 5:**
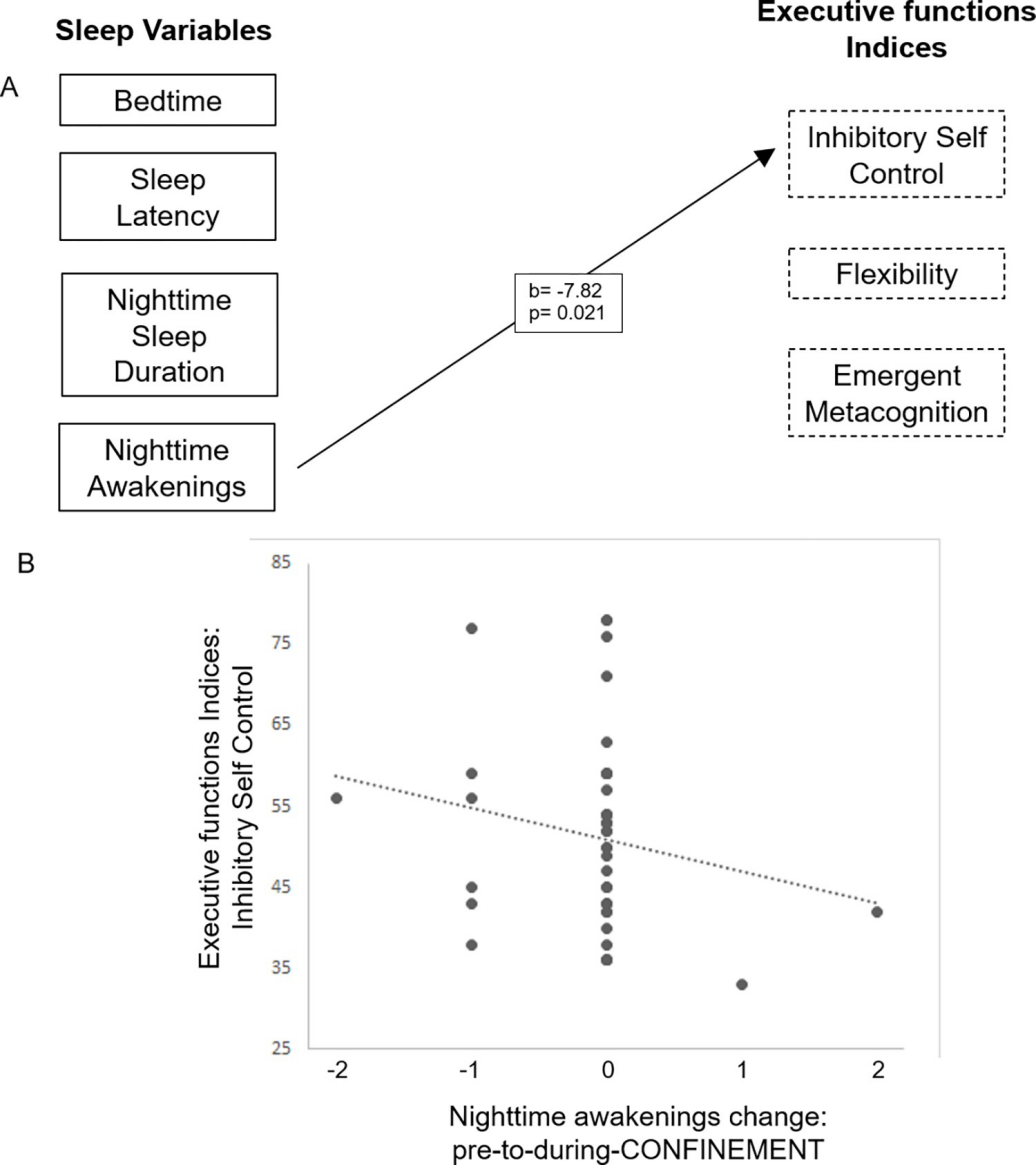
Prediction of executive functions indices by confinement-induced sleep behavior changes in young children. (A): The change in sleep is computed from subtraction pre-to-during-CONFINEMENT in each sleep behavior. Missing values indicate that sleep behavior did not survive the statistical backward selection, b indicates unstandardized beta coefficients, p represents corrected p-values from the linear mixed model. (B) Evolution of the change pre-to-during-CONFINEMENT of Nighttime awakenings and his association with the Inhibitory Self Control Indices. Dashed line automatically fitted with Excel software.

Confinement-induced changes in children’s sleep behavior (*Bedtimes*, *Sleep Latency*, *Nighttime Sleep Duration* and *Nighttime Awakenings*) did not significantly predict the global quantification executive functions as represented by GEC at FOLLOW-UP (S1 Fig). However, specific effects were observed in indices, such that the confinement-induced change in *Nighttime Awakening* negatively predicted later *Inhibitory Self Control* (b = -8.32, p = 0.021, Fig 5). This indicates that children who experienced an increase in *Nighttime Awakenings* due to the confinement demonstrated significantly lower *Inhibitory Self Control* 6 months later. This effect was specific to Inhibition, and no predictive effect was found for indices of *Flexibility* or *Emergent Metacognition*.

**Fig 6:**
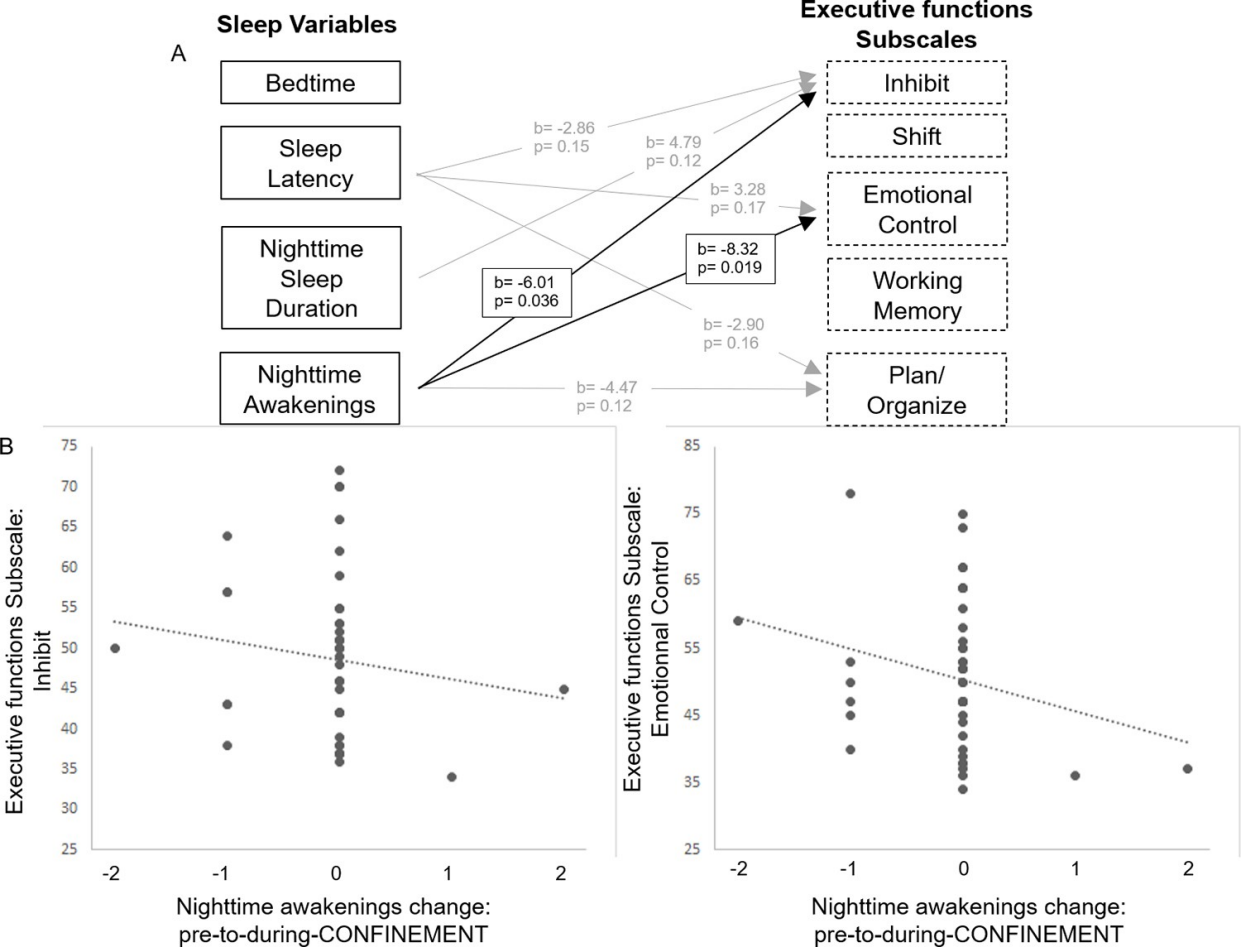
Prediction of executive function subscales by confinement-induced sleep behavior changes in young children. (A)The change in sleep is computed from subtraction pre-to-during-CONFINEMENT in each sleep behavior. Missing values indicate that sleep behavior did not survive the statistical backward selection, b indicates unstandardized beta coefficients, p represents corrected p-values from the linear mixed model. (B) Evolution of the change pre-to-during-CONFINEMENT of Nighttime awakenings and his association with the Inhibit and Emotional Control Subscale. Dashed line automatically fitted with Excel software.

To further detail the specificity of sleep-based predictions of later executive functions, the next analysis focused on the executive function subscales (*Inhibit*, *Shifting*, *Emotional Control*, *Working Memory*, *Planning/Organizing*). We observed an alignment with the relation between *Nighttime Awakening* and *Inhibitory Self Control* index, such that the confinement-induced change in Nighttime awakening also *negatively* predicted the specific subscale Inhibitory self-control at FOLLOW-UP. In other words, more frequent *Nighttime awakenings* were associated with reduced *Inhibit* subscale scores (b = -6.014, p = 0.036, Fig 6). Further, more frequent *Nighttime Awakenings* also showed a negative effect on the *Emotional Control* subscale at FOLLOW-UP (b = -8.32, p = 0.019). No significant effects of the change in *Nighttime Awakening* were found for the remaining subscales *Shift*, *Working Memory*, *or Plan/Organize*. In contrast to *Nighttime Awakenings*, none of the other confinement-related sleep behaviors (*Bedtime*, *Sleep Latency*, or *Nighttime Sleep Duration)* were significant predictors for later executive function subscales (*Inhibit*, *Shift*, *Emotional Control*, *Working Memory*, and *Plan/Organize*).

In summary, this study confirms the hypothesis that sleep behavior can be targeted to protect cognitive developmental processes in preschool children under challenging contextual circumstances. In particular, our data uncovered the specific connection between acutely-increased Nighttime awakening and reduced later Inhibitory self-control outcomes in young children.

## 4. Discussion

This study evidenced the role of sleep behavior in developmental executive function outcomes in preschool children, located in Europe and principally in Switzerland, within an observational-experimental setting. We hypothesized that acute changes in preschool children’s sleep behavior resulting from COVID-19 confinement affect executive functions assessed 6 months later. Core sleep behavior (*Bedtime*, *Sleep Latency*, *Nighttime Sleep Duration*, *Nighttime Awakenings)* and standard scores of executive functions (subscales, indices, composite) were evaluated. Results showed three findings. First, we found that sleep behavior of preschool children changed during the COVID-19 confinement. Second, we evidenced a relationship between sleep behavior and executive function, such that acutely increased *Nighttime Awakenings* predicted lower *Inhibitory Self Control* indices. Third, in line with this finding, increased *Nighttime Awakenings* predicted lower *Inhibit* and *Emotional Control* subscales. Thus, our results confirmed that preschool children’s sleep behavior is a core target to protect cognitive developmental processes under challenging contextual circumstances. Overall, our study provides support to the Sleep-Umbrella-Model proposing that good sleep quality serves as a protective factor for cognitive maturation during childhood development.

We observed that preschool children’s bedtimes became more regular and children fell asleep faster, which confirms earlier observations in young children and adults [19, 20, 71]. However, previous finding that adults experienced prolonged *Nighttime Sleep Duration* [23] was not confirmed in this preschool children cohort.

In contrast with our previous investigation [24], no significant change from pre- to during-CONFINEMENT existed in *Nighttime Sleep Duration* and *Nighttime Awakening*, which is possibly based on the reduced sample size, for which FOLLOW-UP data was obtained (n = 45 vs. n = 412). Yet, interestingly a main effect became apparent in the relation of acutely increased *Nighttime Awakenings* with later reductions in *Inhibitory Self Control*, *Inhibit* and *Emotional Control*. The effect was apparent in several variables presenting a certain consistency of this finding. Crucially, and in alignment with the previous study [24], sleep variables as well as their change, were characterized by inter-individual differences: while some preschool children experienced a "worsening", others showed an "improvement" in sleep quality. While such variability is influenced by family and household context, *e*.*g*., stress [24], physical activity and daylight exposure [72], our present data showed that it nonetheless determines later developmental or cognitive outcomes, which would support a predominant role of sleep quality in behavioral trajectories across development, as presented in the Sleep-Umbrella-Model.

Although sleep quality improved overall from before to during confinement in this subsample, this was not true for all sleep behaviors and all children. Particularly *Nighttime Awakenings* demonstrated increases in some children (although not significant) indicating more fragmented sleep and more fragmented sleep was associated with lower *Inhibitory Self Control*. Our results demonstrate a link between preschool children’s *Nighttime Awakenings* and executive function outcome, involving particularly *Inhibitory Self Control*. Sleep crucially contributes to the maturation of central brain functions in animals [28, 73] and plays a mediating role in human health [48, 49, 74]. The current investigation extends this concept by demonstrating a medium-term effect of young children’s sleep alterations on cognitive maturational status. The link between sleep behavior and *Inhibitory Self Control* is specific to the quality of sleep fragmentation quantified as *Awakenings* from night sleep. In other words, this new finding extends the sleep-neurodevelopment concept with the factor that the acute worsening of nighttime sleep quality at preschool-age relates to later self-regulation outcomes of behavioral inhibition. This also supports our new model concept of sleep as an umbrella for brain protection. These results are supported by observations in preschool children showing a relation between *Nighttime Awakening* and *Inhibitory Self-Control* [25, 74]. Notwithstanding the limited statistical power with the current sample size, a specificity of our results was demonstrated in two ways. First, the effect was specific to *Inhibitory Self-Control*, while no effect was found in the GEC or any other index or subscale. Second, results were specific to the sleep-change induced by the confinement, as demonstrated by a lack of prediction of executive functions outcomes by sleep behaviors before the confinement (retrospective assessment pre-CONFINEMENT). Because the cohort was composed of participants from different countries, data did not allow control for school closure conditions. Nevertheless, the restriction conditions at the two assessment time points were similar among the represented countries, and thus we assume negligible effects on results.

Like the association between sleep and *Inhibitory Self Control*, our findings showed a link between *Inhibit* and *Emotional Control* subscales. This further strengthens the new insight that one specific component of executive functions is predicted by the changes in *Nighttime Awakenings* uniquely while neither GEC nor other subdomains was affected. We thus conclude that the specific event (*i*.*e*., confinement) targeted a unique aspect pertinent to the inhibitory component of executive function.

The observed relationship may result from neuroanatomical connectivity, particularly concerning the amygdala and its role in emotion regulation [75, 76]. The neuroanatomical circuitry of emotional processing matures during the preschool years [77], and executive functions depend on development of the prefrontal cortex [41, 77, 78]. Poor sleep in adults decreases the connectivity among the amygdala, medial prefrontal cortex, and orbitofrontal cortex which negatively affects emotional processing [67]. and emotional control [79, 80]. Similarly, insufficient sleep affects inhibitory behavior and emotion regulation in children [81]. A drastic decrease in sleep quality of young children could thus promote emotional dysfunction through a “weakening” of connections between amygdala and prefrontal cortex. In a chronical state, this may affect inhibitory behavior in the long term, in line with the Sleep-Umbrella-Model.

## 5. Conclusion

Results from this investigation support the role of sleep quality in cognitive function, particularly in the context of development. Sleep behavior and executive function outcomes in preschool children were quantified with an observational-experimental setting during the COVID-19 confinement. Like alterations in sleep behaviors reported for adults, our findings demonstrate that preschool children also experienced altered sleep in the COVID-19 context, which specifically predicted outcomes of inhibitory self-regulation 6 months later. Acute worsening of nighttime sleep during sensitive periods may thus interfere with maturational processes of cognition. Sleep behavior is a target benefiting cognitive developmental processes in preschool children under challenging contextual circumstances.

## Supporting information

S1 Fig Prediction of the global executive function composite GEC by confinement-induced sleep behavior changes in young children. The change in sleep is computed from subtraction pre-to-during-CONFINEMENT in each sleep behavior. No significant association was found between the Sleep Variables and the Executive Function Composite.
(TIF)

S2 Fig Prediction of Indices due to sleep behavior in preschool children based on the pre-CONFINEMENT (before 2020 COVID-19 confinement). Missing values indicate that sleep behavior did not survive the statistical backward selection.
(TIF)

S3 Fig Prediction of Subscales due to sleep behavior in preschool children based on the pre-CONFINEMENT (before 2020 COVID-19 confinement)—Missing values indicate that sleep behavior did not survive the statistical backward selection—(b) refer to unstandardized beta coefficients, and (p) indicates corrected p-values from the linear mixed model. (TIF)

S4 Fig Raw and corrected data of executive functions for subscales (A), indices (B), and GEC (C). Raw values are illustrated in blue, corrected values (T-scores for age or sex, based on standardized table) in red.
(TIF)
