# Supplementary figures and images for: Sleep as a protective factor of children’s executive functions: A study during COVID-19 confinement
Source: PLoS One. 2023 Jan 11;18(1):e0279034. doi: 10.1371/journal.pone.0279034 (PMC9833525; doi:10.1371/journal.pone.0279034)

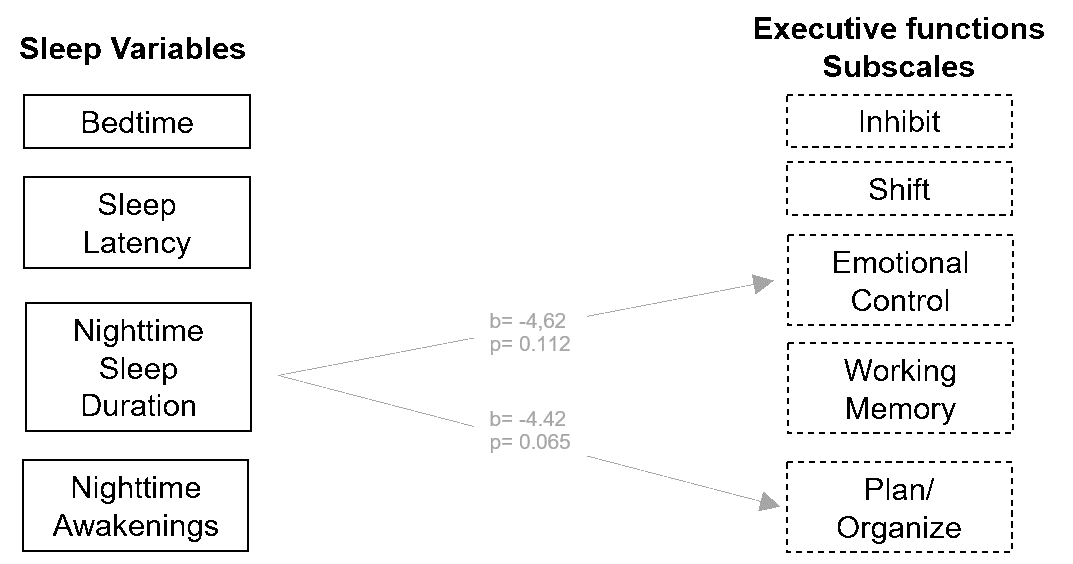

Supplement: S3 Fig — (TIF) [file pone.0279034.s003.tif]

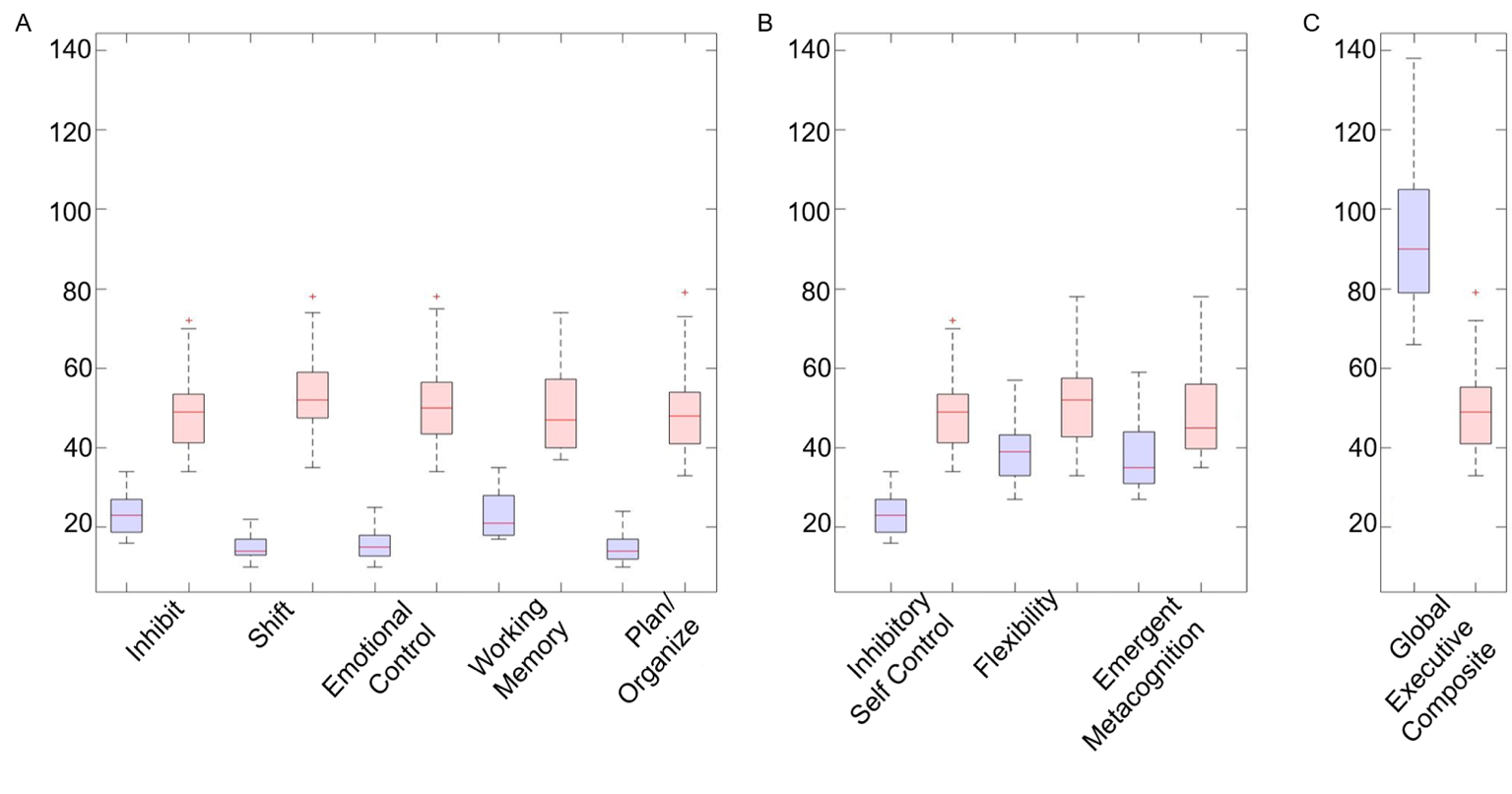

Supplement: S4 Fig — Raw and corrected data of executive functions for subscales (A), indices (B), and GEC (C). Raw values are illustrated in blue, corrected values (T-scores for age or sex, based on standardized table) in red. (TIF) [file pone.0279034.s004.tif]
